# Manual of Bacteriology

**Published:** 1891-06

**Authors:** 


*Manual of Bacteriology.* By EDGAR M. CROOKSHANK, M.B. Third Edition. Pp. 460. London: H. K. LEWIS. 1890.

A hearty word of welcome must be given to the Third Edition of this admirable work. A good deal of new matter has been added, and greater preciseness in several cases has been given to previous studies. The beautiful illustrations continue to be the leading feature of the book.

